# Maintaining the Physiological Lateral Flexion Gap in the Kinematically Aligned TKA Does Not Compromise Clinical Outcomes at One-Year Follow-Up

**DOI:** 10.3390/jcm13123423

**Published:** 2024-06-11

**Authors:** Cristina Jimenez-Soto, Joaquín Moya-Angeler, Vicente J. León-Muñoz, Carlo Theus-Steinmann, Bernhardt Christen, Tilman Calliess

**Affiliations:** 1 Medical School, University of Murcia, 30005 Murcia, Spain; cristinajmsoto@gmail.com; 2 Department of Orthopaedic Surgery and Traumatology, Hospital General Universitario Reina Sofía, 30003 Murcia, Spain; vleonmd@gmail.com; 3 Instituto de Cirugía Avanzada de la Rodilla (ICAR), 30005 Murcia, Spain; 4 Articon Spezialpraxis für Gelenkchirurgie, Berner Prothetikzentrum, 3013 Bern, Switzerland; c.theus@articon.ch (C.T.-S.); b.christen@articon.ch (B.C.); t.calliess@articon.ch (T.C.)

**Keywords:** gap balancing, flexion gap, kinematic alignment, total knee arthroplasty, robotic-assisted, MAKO, individualised alignment

## Abstract

**Background:** Instability is a common cause of (total knee arthroplasty) TKA failure, which can be prevented by achieving proper gap balance during surgery. There is no consensus on the ideal gap balance in TKA, and different alignment philosophies result in varying soft-tissue tightness. Traditional TKA aims for symmetric compartment balance, while kinematic alignment (KA) restores anatomy and accepts asymmetric flexion gaps. This study evaluated the impact of these philosophies on the flexion gap balance and clinical outcomes. **Methods:** A retrospective review of 167 patients who received true or restricted KA robotic-assisted TKA with at least one year of follow-up was conducted. The groups were based on intraoperative flexion gap differences: symmetric (0–1 mm) (n = 94) and asymmetric (2–5 mm) (n = 73). **Results:** Preoperative demographics and postoperative clinical and functional scores were compared. Both groups were similar in demographics and preoperative scores. True KA alignment was more likely to result in an asymmetric flexion gap, while restricted KA produced symmetric gaps. **Conclusions:** The study found no adverse effects from the physiological asymmetric flexion gap, with clinical and functional outcomes comparable to symmetric gaps. A 5 mm difference between the medial and lateral gap width did not negatively impact the outcomes. True KA more frequently results in a physiological asymmetric flexion gap.

## 1. Introduction

Total knee arthroplasty (TKA) is well-established as a cost-effective procedure, enabling a higher quality of life for patients with advanced osteoarthritis (OA) and presenting a high success rate [1,2,3,4]. However, this technique is not exempt from complications, with the instability of the tibiofemoral joints accounting for 10 to 20% of TKA revisions [5]. The unbalance in contact forces during load-bearing activities may lead to femoral condyle lift-off, paradox femorotibial kinematics, increased polyethylene wear [6], and pain. One cause of instability in TKA is ligamentous imbalance [7,8]. During surgery, the gap balance technique aims to achieve the correct alignment and soft-tissue balance of the knee [9]. In response to mechanical soft-tissue forces, the joint has a gap during flexion and extension that separates the femur and tibia [9,10,11]. Depending on the individual’s anatomy and soft-tissue restrictions, this gap may differ in the medial and lateral compartments [9]. No consensus exists regarding the correct gap balancing needed to stabilise the knee joint in TKA [10,12,13]. Nevertheless, the traditional approach has been to achieve symmetry between the medial and lateral compartments when the knee is in flexion and extension [11,14,15]. However, the normal knee shows some gap imbalance in flexion between the medial and lateral compartments [16]. This has led some authors to claim that achieving an asymmetrical flexion gap could lead to superior outcomes compared to a symmetric gap [10,12,13,17]. In contrast, others declare no benefit of one over the other [15,18]. Over the past few decades, techniques for implanting TKA have evolved towards achieving a more customised alignment of the components, resulting in improved functional outcomes and patient satisfaction [19]. In particular, computer- and robotic-assisted technologies enable the control of the three-dimensional component position and help to objectify the femorotibial gaps [9]. However, in this context, new alignment philosophies, such as inverse kinematic alignment or functional alignment, are still based on the principle of equal and symmetric flexion and extension gaps [19,20,21]. This contrasts with the kinematic alignment (KA) idea, which aims to align the knee prosthesis components to closely restore the patient’s constitutional and prearthritic anatomy and thus achieve more natural soft tissue tension and kinematics of the patient’s knee [19,20]. Therefore, the balance in true KA should reflect an asymmetric flexion gap (with more significant natural laxity in the lateral compartment) rather than the symmetric flexion gap [9,10,11,13,17]. This idea is only abandoned when adaptions to the component alignment are made in the restricted KA (rKA) concept to avoid overall knee alignment outside a previously defined range [19,20]. Until today, very limited data are available on the effect of different individualised alignment philosophies on the flexion gap balance and the resulting effect on the clinical outcome. In this context, this study aims to evaluate the influence of a true KA and rKA approach on the resulting flexion gap balance as well as to compare the clinical and functional outcomes of symmetric versus asymmetric flexion gaps in robotic-assisted (RA) TKA.

## 2. Materials and Methods

This was a retrospective study in a consecutive series of 167 patients (true KA or rKA) presenting to a single centre that underwent robotically assisted TKA using the MAKO robotic platform and Triathlon posterior-stabilised implant (Stryker, Mahwah, NJ, USA) between April 2019 and April 2022 for primary knee osteoarthritis. Two senior surgeons performed all surgeries.

The surgeons preplanned all cases based on the proprietary software on the preoperatively segmented computed tomography scan (CT-scan) data [22,23,24]. First, the femoral component was adjusted following the KA principle, setting the distal and posterior resection level to 6 mm to restore the native joint line with the 8 mm thick prosthesis (a 6 mm bone resection based on the CT scan plus 2 mm of cartilage thickness = 8 mm total resection volume) [17,20,25]. The boundaries for KA on the femur were a maximum of a 3° valgus angle and a resulting relative internal rotation of the femur concerning the native trochlea orientation. In cases where the primary implant position based on the distal and posterior resection thickness was outside these boundaries, adjustments were made to a rKA philosophy as follows: the medial distal and posterior femur resections were always left at 6 mm, and only the lateral ones were adapted (less resection) so that the medial column was always reconstructed anatomically following the KA principle [17,20,25]. On the tibia, only a conservative precut was planned at a 4 to 5 mm resection level, with a conservative tibia varus angle between 0° and 2° and a conservative tibia slope ranging from 0° to 3° to best match the native anatomy [17,20]. As a definition, a symmetric medial and lateral femoral resection thickness in flexion and extension was assigned to the “true KA” collective, and whenever the resection thickness had more than a 1 mm difference, the case was named “restricted KA” [20].

During surgery, the positioning of the arrays, surgical approach, and bone registration were executed according to the in-house surgical standard and the MAKO standard procedure [26]. First, in surgery, after bone registration, the correct femoral resection level was verified by mapping the remaining cartilage level in the areas without osteoarthritic wear. Minor adjustments were made to the preplanning to best meet the native joint surface. After that, all femoral bone cuts plus the tibia precut were conducted with the assistance of a haptic robotic arm [26]. Then, all osteophytes were meticulously removed with the help of image-based navigation. As in the concept of KA, no classic soft tissue releases were performed except for the posterior cruciate ligament resection for the PS prosthesis [20]. After these steps, the flexion and extension gaps were recorded with the help of the Stryker Monogram Knee Balancer (Stryker, Mahwah, NJ, USA) and the MAKO ligament balancing software tool [26]. The target zone for the gap balance was a symmetric extension gap of 18–20 mm in height (±1 mm difference between the medial to lateral sections was accepted).

In cases of medial tightness, the tibia orientation was aligned in more varus and recut with a maximum of 5° tibial varus. In cases of remaining medial tightness, the tibia plateau was downsized by one size and aligned to the very lateral border of the plateau. The medial overhanging bone was resected to indirectly release the medial joint space. In cases of lateral tightness, the tibia was adjusted to be more valgus to a maximum of 1° valgus for the overall limb alignment (thus, depending on the femur orientation) [17,19,20,25]. Releases on the iliotibial band, lateral capsule, or femoral insertion of the lateral collateral ligament were performed in cases with remaining lateral tightness to achieve a symmetric extension gap. In the symmetric tightness of the extension gap, the tibia resection level was increased to meet the target zone. The flexion gap target was an isometric balance medially to the extension gap of ±2 mm, whereas laterally, the natural laxity up to a 5 mm difference from the medial was accepted. Thus, no changes in the femoral rotation were made to balance the flexion gap. After these adaptions, the virtually planned adjusted tibia position was recut with robotic assistance. This situation was then evaluated with a trial prosthesis to ensure full (0°) extension and a symmetrically balanced extension gap. In flexion, equal medial stability was tested and recorded, as well as lateral laxity [17,20,25]. When satisfactory knee balance and range of movement were achieved, the prosthesis was positioned accordingly.

For data analysis, the medial and lateral flexion gaps were recorded at the end of surgery. The difference between the lateral and medial flexion gaps was calculated for statistical analysis.

In addition to the intraoperative surgical data, baseline patient demographics (age, gender, surgical side, ASA score, and body mass index (BMI)) were recorded and analysed using descriptive statistics. Preoperative and one-year postoperative clinical and functional outcomes were collected; these included the range of motion (ROM), Knee Society score (KSS), Oxford knee score (OKS), knee injury and osteoarthritis outcome score (KOOS), and EQ-D5. In addition, the forgotten joint score (FJS12) was included at the one-year follow-up.

For data analysis, the groups were established based on the recorded intraoperative flexion gap (mediolateral) differences: symmetric (0–1 mm) (n = 94) versus asymmetric group (2–5 mm) (n = 73). We calculated the sample size for adequate power, accepting an alpha risk of 0.05 and a beta risk of 0.2 in a bilateral contrast. The sample of our series was sufficient for adequate statistical power.

We used the Statistical Package for the Social Sciences (SPSS), version 28 for Windows (SPSS, Inc., Chicago, IL, USA) to perform the statistical analysis. We used the Mann–Whitney U and Chi-square tests to assess the differences between the group variables (based on the millimetres of the mediolateral flexion gap difference). We used the Kolmogorov–Smirnov test to check that the *p*-values were above the significance level of 0.05, with the null hypothesis that the data fitted a normal distribution rejected.

## 3. Results

The descriptive characteristics of the variables of the two groups are shown in Table 1. There were no significant differences between the groups regarding age at surgery, BMI, morbidity state (ASA), or surgical side. Female patients were overrepresented in the symmetric group (*p* < 0.05).

**Figure 1:**
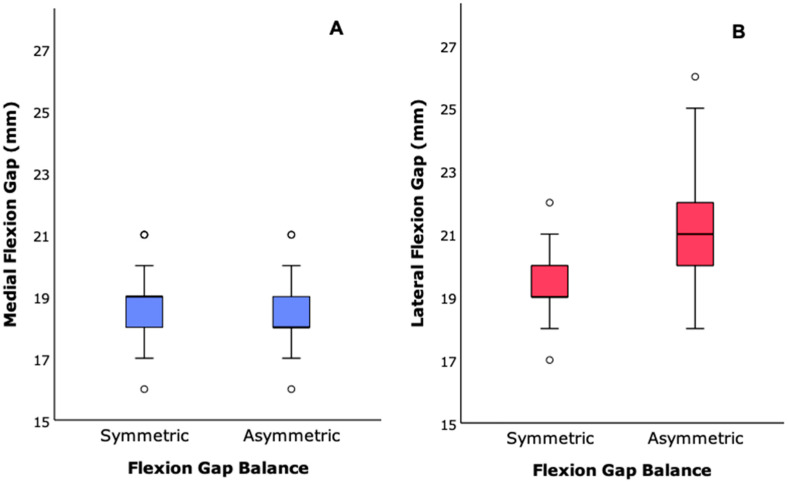
Box plot of the study groups’ (**A**) medial and (**B**) lateral flexion gaps with trials (in mm).

The mean joint gaps showed significant differences between the groups, as shown in Table 2, with the gap for the medial compartment in flexion being more significant in the symmetric group. In contrast, the lateral flexion gap was more significant in the asymmetric flexion gap group, as shown in Figure 1 (*p* < 0.05). For the asymmetric group, the mean difference between the medial and lateral gap was 2.2 mm, with a range from 2 to 5 mm.

There were no significant differences in the clinical outcomes (*p* > 0.05), as illustrated in Table 3.

No differences were observed (*p* > 0.05) in the postoperative functional outcome parameters, as shown in Table 4.

**Figure 2:**
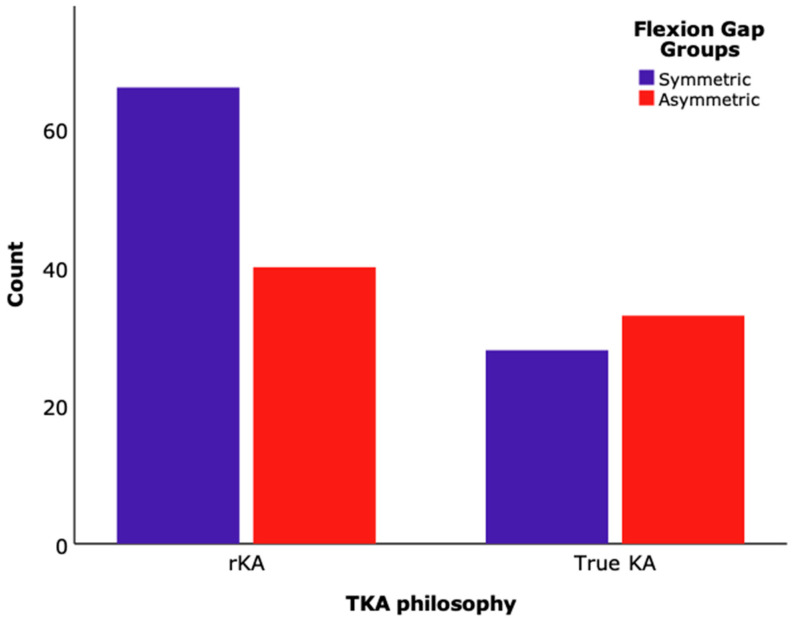
Bar chart representing the number of patients (count) for restricted KA (rKA) or actual KA (KA) in each flexion gap balance group: symmetric (0 to 2 mm) and asymmetric (2 to 5 mm).

Patients who were positioned following the true KA principle were more likely to generate an asymmetric flexion gap balance (2–5 mm) than rKA (*p* < 0.05), as shown in Table 5 and Figure 2.

## 4. Discussion

Our study’s findings add significant insights to the ongoing debate over the optimal approach for gap balancing in TKA. Our results indicate that (1) true KA results in a more physiological flexion gap balance and already a rKA philosophy leads to a more symmetrical flexion gap (Table 5 and Figure 2); and (2) there were no disadvantages of asymmetric flexion gaps up to a 5 mm difference between the medial and lateral gap over the classical symmetric gap in TKA, with both groups showing similar outcomes (Table 3 and Table 4). This article highlights the impact of different alignment approaches on flexion gap balance in total knee arthroplasty (TKA) and their clinical outcomes. It compares the traditional method aiming for symmetric balance with the kinematic alignment (KA) approach, which accepts natural asymmetry to restore native knee anatomy. By reviewing 167 patients, the study found that an asymmetric flexion gap of up to 5 mm in KA does not negatively affect the clinical outcomes, suggesting that respecting natural knee anatomy may improve TKA stability and success. The real importance of this trial lies in its potential to guide surgical practices towards more individualised, anatomy-respecting techniques, thereby enhancing patient outcomes and reducing the risk of TKA failure due to instability.

The mechanical alignment criteria used by most surgeons until recent decades have sought a symmetrical gap between the medial and lateral compartments during flexion and extension to ensure the stability and longevity of knee implants [18,19]. Minoda et al. [27] demonstrated that the asymmetry between the flexion and extension gaps increased polyethylene wear. Chia et al. [11] established that patients with imbalanced gaps have poorer clinical outcomes at six months than those with balanced flexion and extension joint gaps. Romero et al. [10] observed that increased lateral flexion laxity is associated with poorer clinical outcomes.

However, it is essential to note that the native knee has some degree of asymmetry in natural laxity between the two compartments in flexion [28,29,30]. As flexion increases, the lateral joint space opens by an average of 6.7°. A so-called “lift-off” phenomenon occurs, whereby the lateral condyle detaches from the lateral tibial plate by about 2.1 mm, both in and out of load [16,31,32]. This asymmetry can be explained by the physiological laxity of the lateral collateral ligament in flexion and by the morphology of the joint itself [28]. Present philosophies, such as KA, advocate for a more anatomically, physiologically, and personalised approach, recognising the inherent asymmetries in individual knee anatomy and mechanics [19,20]. Some studies have shown an association between an asymmetry in the joint gaps and better outcomes after TKA. Meneghini et al. [13] proved that the clinical outcomes were superior for patients with lateral laxity in flexion. Higuchi et al. [33] observed that the knee flexion angle improves when the flexion gap is larger than the extension gap.

The degenerative changes in knee OA impact the joint’s anatomy. While the changes primarily impact the cartilage, there may be some degree of soft-tissue imbalance in the form of instability, deformity, or contracture [12,18,28,30]. When considering that the soft-tissue imbalances in the arthritic knee could be a cause of the disease, it seems reasonable that TKA techniques aim to achieve proper soft-tissue balance and alignment. However, no consensus exists regarding the correct value to which one should aim for gap balancing [13,14,27]. The traditional approach for the gap balancing technique has been to achieve symmetrical extension and flexion gaps [14]. However, no studies have quantified what is considered a symmetric and asymmetric value. In prior investigations, the gap balance is considered asymmetric when a difference is greater than 2 mm [11,15]. In our study, the target was the same value obtained for the extension gap of ±2 mm for the medial compartment and up to ±5 mm for the lateral compartment (Table 2). This small difference accounts for slight variations in the normal knee soft-tissue tension, which physiologically exerts some degree of individual variability to allow for the normal functioning of the knee [16].

The results of our study demonstrate an association between KA techniques and gap balance in flexion, with actual KA being more likely to generate a physiological asymmetric flexion compartment. Similar clinical and functional outcomes to a symmetric flexion gap balance are observed. Thus, the dogma of a symmetrical flexion gap can be questioned as mandatory for successful TKA, at least when the individual joint surface is closely reconstructed.

A subtle balance between stability and mobility orchestrates the normal functioning of the knee. Anatomically, the knee presents more contact in the medial compartment than in the lateral, suggesting that the centre of rotation of the knee is in the medial compartment [28,29,30,32]. Consequently, more significant laxity in the lateral compartment [9,13,34] would enable the physiologic posterolateral femoral roll-back [13,21]. Thus, it seems reasonable that some authors [13,21,33] observed better outcomes when a certain degree of asymmetry was present.

Achieving perfect soft-tissue balance during TKA is difficult, so a 0 to 2 mm difference between the medial and lateral compartments is still considered symmetrical [11,15,21]. It is plausible that when restoring constitutionality, there will be inequalities in the joint gaps through the medial and lateral compartments, with a more significant lateral gap than the medial during flexion. Therefore, some authors have postulated that a slight asymmetry in the gap balance may be acceptable [10,11,13,17,21]. Our findings support this statement, with no significant differences between the symmetric (0–2 mm) and asymmetric (2–5 mm) flexion gaps in knee function and clinical scores.

To cater to the diverse needs of patients and the complexities of knee OA, the concept of KA has evolved, leading to the development of different techniques, such as actual or unrestricted KA and rKA [19]. KA seeks to replicate the patient’s predisease native knee anatomy and alignment precisely. It involves adjusting the components to match the patient’s individual alignment and joint line orientation without any predetermined alignment targets different from each patient’s own constitutional and individual axes. On the other hand, rKA adjusts the knee components to match the patient’s anatomy closely but with predefined safe zones to avoid excessive deviation from the mechanical axis [19,20]. The choice between KA and rKA often depends on individual factors, such as the degree of deformity, ligament stability, and overall alignment of the lower limb [19,20]. Thus, it seems reasonable that rKA is associated with a symmetrical gap balance in our cohort of patients. In contrast, the freedom in actual KA allows for a more asymmetrical gap balance (Table 5 and Figure 2).

Robotic-assisted surgery can predict knee implant laxity before making femoral resections to achieve an optimal implant position, alignment, and soft-tissue balance [9,26]. Laxity in the native knee has been shown to increase significantly from 0° to 45° but then remain constant or increase only slightly at >90° of flexion [9]. Recording the laxity and stability throughout the entire ROM while applying controlled ligament tension may lead to the achievement of a more natural and individualised gap balance [26].

There are some limitations to our study. First, a control group in which mechanically aligned TKA was performed was absent. Second, our study was a retrospective study in a consecutive series of patients, with the methodological limitation that this may represent. Third, follow-up was only one year. Lastly, only one implant design was utilised (posterior-stabilised implant), and other implant designs could yield different outcomes, as the resection of the posterior cruciate ligament may increase the flexion gap [2,12].

## 5. Conclusions

True KA is more likely to generate a physiological asymmetric flexion gap with up to 5 mm lateral laxity than rKA. Both philosophies yield similar clinical and functional outcomes. Thus, the traditional approach of aiming for symmetric flexion compartments can be questioned, and the knee gap balance can be adapted within a physiological range to cater to each patient’s individual needs. However, long-term results are still lacking in this context.

## Figures and Tables

**Table 1:** Baseline characteristics of the study groups. Patients were divided into two groups based on their mediolateral flexion gap difference: symmetric (0 to 2 mm) and asymmetric (2 to 5 mm). Values are shown as the ^a^ mean and standard deviation or ^b^ n and (%). All differences were considered significant at a probability level of 95% (*p* < 0.05).

		Symmetric Group (n = 94)	Asymmetric Group (n = 73)	*p*-Value
Gender ^b^	Female	66 (70.2%)	36 (49.3%)	0.006
	Male	28 (29.8%)	37 (50.7%)	
ASA ^b^	ASA I	9 (9.6%)	2 (2.7%)	0.055
	ASA II	66 (70.2%)	46 (63%)	
	ASA III	19 (20.2%)	24 (32.9%)	
Side ^b^	Left	49 (52.1%)	27 (37%)	0.051
	Right	45 (47.9%)	46 (63%)	
Age ^a^		69.96 (8.49)	70.47 (7.3)	0.969
BMI (kg/m^2^) ^a^		28.32 (11.49)	30.95 (19.79)	0.084

Baseline characteristics of the study groups: ASA: American Society of Anesthesiologists physical status classification (ASA I: normal healthy patient; ASA II: patient with mild systemic disease, and ASA III: patient with severe systemic disease); BMI: body mass index; values are shown as the mean and standard deviation or b n and (%). All differences were considered significant at a probability level of 95% (*p* < 0.05).

**Table 2:** The study groups’ joint balance with trials (in mm).

		Symmetric (n = 94)	Asymmetric (n = 73)	*p*-Value
Gap balance with trials (mm)	Medial extension	18.86 (1)	18.84 (1.04)	0.203
	Medial flexion	18.84 (0.92)	18.52 (0.87)	0.007
	Lateral extension	19.04 (0.98)	19.06 (1.03)	0.292
	Lateral flexion	19.17 (0.93)	21.40 (1.53)	<0.001

**Table 3:** Preoperative and postoperative clinical outcomes.

		Symmetric (n = 94)	Asymmetric (n = 73)	*p*-Value
Preoperative	Mean KOOS	37.88 (13.48)	37.63 (14.48)	0.879
	Oxford knee score	24.26 (6.69)	23.59 (7.36)	0.515
	Knee Society Score	99.54 (20.56)	96.73 (17.38)	0.135
	EQ-5D score	0.6 (0.24)	0.56 (0.26)	0.349
Postoperative	Mean KOOS	77.51 (20.70)	77.91 (17.80)	0.838
	Oxford knee Score	40.46 (9.80)	40.07 (7.68)	0.246
	Knee Society score	189.6 (17.24)	184.92 (29.10)	0.689
	EQ-5D score	0.85 (0.22)	0.84 (0.22)	0.779
	FJS-12	66.85 (28.71)	61.82 (30.26)	0.298

KOOS: knee injury and osteoarthritis outcome score. FJS-12: forgotten joint score.

**Table 4:** Preoperative and postoperative functional parameters among the groups. Values are shown as the ^a^ mean and standard deviation or ^b^ n and (%).

			Symmetric (n = 94)	Asymmetric (n = 73)	*p*-Value
Preoperative	Active extension ^a^		−3.83 (15.11)	−2.88 (13.59)	0.189
	Active flexion ^a^		117.66 (18.48)	119.73 (20.21)	0.267
	Active ROM ^a^		113.83 (15.72)	116.85 (16.51)	0.161
	Standing alignment ^b^	Neutral	25 (26.6%)	15 (20.5%)	0.078
		Valgus	23 (24.5%)	10 (13.7%)	
		Varus	46 (48.9%)	48 (65.8%)	
	Anteroposterior stability ^b^	<5	79 (84.0%)	58 (79.5%)	0.690
		5–10	15 (16.0%)	13 (17.8%)	
	Mediolateral stability ^b^	<6	72 (76.6%)	50 (68.5%)	0.292
		6–9	15 (16.0%)	19 (26.0%)	
		9–14	6 (6.4%)	4 (5.5%)	
Postoperative	Active extension ^a^		−0.05 (2.01)	1.51 (19.96)	0.485
	Active flexion ^a^		128.78 (7.68)	119.73 (20.21)	0.410
	Active ROM ^a^		128.72 (8.13)	116.85 (16.51)	0.582
	Standing alignment ^b^	Neutral	80 (85.1%)	60 (82.2%)	0.891
		Valgus	6 (6.4%)	6 (6.4%)	
		Varus	8 (8.5%)	6 (8.2%)	
	Anteroposterior stability ^b^	<5	86 (91.5%)	70 (95.9%)	0.255
		5–10	8 (8.5%)	3 (4.1%)	
	Mediolateral stability ^b^	<6	89 (94.7%)	65 (89.0%)	0.177
		6–9	5 (5.3%)	8 (11.0%)	
		9–14	0 (0%)	0 (0%)	

ROM: range of motion.

**Table 5:** Number of patients from each group according to TKA philosophy. Values are shown as n and %.

	Symmetric (n = 94)	Asymmetric (n = 73)	*p*-Value
True KA	28 (29.8%)	33 (45.2%)	0.040
Restricted KA	66 (70.2%)	40 (54.8%)	

## Data Availability

Data are contained within the article.
